# Significance of p53-binding protein 1 nuclear foci in uterine cervical lesions: endogenous DNA double strand breaks and genomic instability during carcinogenesis

**DOI:** 10.1111/j.1365-2559.2011.03963.x

**Published:** 2011-09

**Authors:** Katsuya Matsuda, Shiro Miura, Tomomi Kurashige, Keiji Suzuki, Hisayoshi Kondo, Makoto Ihara, Hisayoshi Nakajima, Hideaki Masuzaki, Masahiro Nakashima

**Affiliations:** 1 Department of Tumour and Diagnostic Pathology, Atomic Bomb Disease Institute, Nagasaki University Graduate School of Biomedical SciencesNagasaki, Japan; 2 Department of Obstetrics and Gynaecology, Nagasaki University HospitalNagasaki, Japan; 3 Tissue and Histopathology Section, Division of Scientific Data Registry, Atomic Bomb Disease Institute, Nagasaki University Graduate School of Biomedical SciencesNagasaki, Japan; 4 Department of Radiation Medical SciencesNagasaki, Japan; 5 Biostatics Section, Division of Scientific Data RegistryNagasaki, Japan; 6 Department of Radioisotope Medicine, Atomic Bomb Disease Institute, Nagasaki University Graduate School of Biomedical SciencesNagasaki, Japan; 7 Nagasaki University School of Health SciencesNagasaki, Japan

**Keywords:** 53BP1, DNA damage response, genomic instability, immunofluorescence, uterine cervical cancer

## Abstract

**Aims:**

A defective DNA damage response can result in genomic instability (GIN) and lead to transformation to cancer. As p53-binding protein 1 (53BP1) localizes at the sites of DNA double strand breaks (DSBs) and rapidly forms nuclear foci (NF), the presence of 53BP1 NF can be considered to be an indicator of endogenous DSBs reflecting GIN. Our aim was to analyse the presence of DSBs by immunofluorescence for 53BP1 expression in a series of cervical lesions, to evaluate the significance of GIN during carcinogenesis.

**Methods and results:**

A total of 80 archival cervical tissue samples, including 11 normal, 16 cervical intraepithelial neoplasia (CIN)1, 15 CIN2, 24 CIN3 and 14 squamous cell carcinoma samples, were analysed for 53BP1 NF, human papillomavirus (HPV) infection, and p16^INK4a^ overexpression. The number of 53BP1 NF in cervical cells appeared to increase with progression during carcinogenesis. The distribution of 53BP1 NF was similar to that of the punctate HPV signals as determined by *in-situ* hybridization and also to p16^INK4a^ overexpression in CIN, suggesting an association with viral infection and replication stress.

**Conclusions:**

Immunofluorescence analysis of 53BP1 expression can be a useful tool with which to estimate the level of GIN. During cervical carcinogenesis, GIN may allow further accumulation of genomic alterations, causing progression to invasive cancer.

## Introduction

Any genotoxic agents can induce DNA damage and consequently result in oncogenic alterations. The importance of the DNA damage response (DDR) pathway in tumour suppression is well recognized. A defective DDR can result in genomic instability (GIN), which is generally considered to be central to any carcinogenic process.^1^,^2^ Alternatively, the presence of an activated DDR can be a hallmark of GIN, which may subsequently enhance the carcinogenic process.

The p53-binding protein 1 (53BP1) belongs to a family of evolutionarily conserved DDR proteins with C-terminal BRCA1 C-terminus domains.^3^,^4^ 53BP1 is a nuclear protein that rapidly localizes at the sites of DNA double strand breaks (DSBs) and activates p53 along with other kinases.^5^–^10^ Activated p53 plays critical roles in the DDR, such as cell cycle arrest, DNA repair, and apoptosis.^11^,^12^ It has been well documented *in vitro* with immunofluorescence that 53BP1 exhibits diffuse nuclear staining in untreated primary cells, whereas, after exposure to radiation, 53BP1 localizes at the sites of DSBs and forms discrete nuclear foci (NF).^5^,^6^,^13^,^14^ We have recently demonstrated that, with an immunofluorescence method, 53BP1 NF may serve as a valuable molecular marker of GIN during carcinogenesis.^15^,^16^ GIN seems to be induced at the precancerous stage during thyroid and skin carcinogenesis, as follicular adenoma and actinic keratosis show occasional 53BP1 NF.^15^,^16^ Given that one manifestation of GIN is the induction of endogenous DDR,^17^ we propose that immunofluorescence analysis of 53BP1 expression can be a useful tool with which to estimate the level of GIN as well as the malignant potential of human tumours.

Uterine cervical cancers are believed to develop through a multistep process. Furthermore, it is well established that persistent infections with high-risk human papillomavirus (HR-HPV) represent a necessary cause of high-grade premalignant lesions and subsequent invasive cancer of the uterine cervix. The HR-HPV viral oncogenes, *E6* and *E7*, have been shown to be the main contributors to the development of human papillomavirus (HPV)-induced cervical cancer, and increased expression resulting from integration of the viral DNA into the host genome has been detected in invasive cancers and a subset of high-grade lesions.^18^ It has been shown that *E6* and *E7* together cause polyploidy soon after they are introduced into cells, so GIN is thought to play an essential role in the cellular transformation of cervical epithelium during carcinogenesis. The most manifest function of the E6 protein is to promote the degradation of p53 through its interaction with a cellular protein, E6-associated protein, an E3 ubiquitin ligase.^19^ E7 is known to bind to the retinoblastoma tumour suppressor gene product, retinoblastoma protein (pRb). Phosphorylation of pRb by G_1_ cyclin-dependent kinases releases E2F, leading to cell cycle progression into the S phase. E7 is able to bind unphosphorylated pRb, and this may induce cells to prematurely enter the S phase by disrupting pRb–E2F complexes. One cyclin-dependent kinase inhibitor, p16^INK4a^, which prevents the phosphorylation of pRb family members, is overexpressed when pRb is inactivated by E7.^20^ Normally, overexpression of p16^INK4a^ results in cell cycle arrest, but with E7 expression, this is overcome. Thus, overexpression of p16^INK4a^ has been suggested as a useful biomarker for evaluating HPV pathogenic activity in cervical lesions.

The present study analysed the presence of endogenous DSBs by immunofluorescence for 53BP1 expression in a series of cervical tissues from patients to evaluate the significance of GIN and its association with HPV infection and p16^INK4a^ overexpression during cervical carcinogenesis. Like other tumours, GIN was shown to be induced in cervical epithelium at a precancerous stage, and increased significantly with progression to cancer.

## Materials and methods

### Cervical Lesions

Eighty archival uterine cervical tissue samples were selected for this study from the archives of the Department of Obstetrics and Gynaecology, University Hospital. Accuracy of diagnosis was confirmed by a gynaecological pathologist (H.N.) and a general pathologist (M.N.). Histologically, the 80 primary cervical tissue samples were as follows: 11 normal cervical tissue samples from uteri that were surgically resected because of leiomyoma; and 16 cervical intraepithelial neoplasia (CIN)1, 15 CIN2, 24 CIN3 and 14 squamous cell carcinoma (SCC) samples. All samples were formalin-fixed and paraffin-embedded tissues, from which sections were prepared for immunofluorescence and *in-situ* hybridization (ISH) studies.

### ISH to Detect HR-HPV

ISH was performed with the GenPoint Catalyzed Signal Amplification System (Dako, North America, Inc., Carpinteria, CA, USA) for HR-HPV (types 16, 18, 31, 33, 35, 39, 45, 51, 52, 56, 58, 59, and 68; code Y1443), according to the manufacturer's protocols. After deparaffinization and rehydration, the sections were incubated in 0.8% pepsin for 10 min at 37°C and treated with proteinase for 5 min at room temperature. Sections were immersed in 0.3% H_2_O_2_ in methanol for 20 min, and denatured and hybridized in a humidified chamber at 37°C for 60 min. Detection of hybridized probe was performed with the GenPoint Tyramide Signal Amplification System for Biotinylated Probes (Dako North America, Inc.), with application of primary peroxidase-conjugated streptavidin, biotinyl-tyramide secondary peroxidase-conjugated streptavidin, and the chromogenic substrate diaminobenzidine (DAB). The slides were counterstained with haematoxylin. Evaluation of the HPV ISH signals was performed according to the criteria described previously.^21^–^23^ In brief, the patterns were as follows: (i) a diffuse pattern representing episomal HPV that correlates with viral replication; (ii) a punctate pattern consisting of one or a few discrete signals in the nucleus, indicating HPV integration into the cellular genome; and (iii) a mixed pattern with separate areas containing only integrated or episomal copies, and areas where the integrated virus was hidden in episomal HPV copies.

### Immunofluorescence for 53BP1 Expression

After antigen retrieval with microwave treatment in citrate buffer, deparaffinized sections were preincubated with 10% normal goat serum. Tissue sections were then reacted with anti-53BP1 rabbit polyclonal antibody (Bethyl Labs, Montgomery, TX, USA) at a 1:200 dilution. The slides were subsequently incubated with Alexa Fluor 488-conjugated goat anti-rabbit antibody (Invitrogen, Carlsbad, CA, USA). Specimens were counterstained with 4′,6-diamidino-2-phenylindole dihydrochloride (DAPI-I; Vysis, Downers Grove, IL, USA), analysed, and photographed with a High Standard All-in-One Fluorescence Microscope (Biorevo BZ-9000; Keyence Japan, Osaka, Japan). Signals were analysed at a ×1000 magnification.

### Evaluation of Immunofluorescence Results

**Figure 1:**
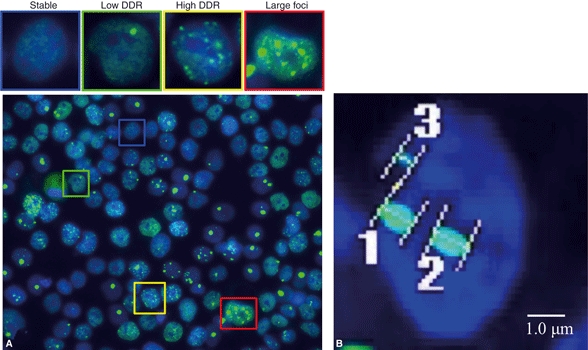
**A**, four types of p53-binding protein 1 (53BP1) expression found in HeLa cells: (1) stable type – faint and diffuse nuclear staining; (2) low DNA damage response (DDR) type – one or two discrete nuclear foci (NF); (3) high DDR type – three or more discrete NF; and (4) large NF type – discrete NF that are larger than 1.0 μm. **B**, measurements of the size of NF in a cell by use of a High Standard All-in-One Fluorescence Microscope (Biorevo BZ-9000; Keyence Japan). 1, 1.01 μm; 2, 1.05 μm; 3, 0.53 μm.

A human cervical cancer cell line, HeLa, was also analysed by immunofluorescence for 53BP1 expression. HeLa cells were cultured in RPMI-1640 medium supplemented with 10% fetal bovine serum, and grown at 37°C in a 5% CO_2_/95% air environment. After fixation with methanol and preincubation with 10% normal goat serum, cultured cells were treated according to the same immunofluorescence method as mentioned above. The pattern of 53BP1 immunoreactivity was classified into four types (Figure 1): (1) stable type – faint and diffuse nuclear staining; (2) low DDR type – one or two discrete NF; (3) high DDR type – three or more discrete NF; and (4) large NF type – discrete NF that are larger than 1.0 μm. The percentage of cervical epithelial cells expressing each type of 53BP1 immunoreactivity was calculated in each cervical lesion.

### Dual Immunofluorescence for 53BP1 Expression and ISH to Detect HR-HPV

To assess the association between localization of 53BP1 NF and HR-HPV integration into the host genome in neoplastic cells, dual immunofluorescence for 53BP1 expression and ISH to detect HR-HPV were performed on the same tissue sections. Each section was first labelled by immunofluorescence, subjected to ISH as mentioned above, and photographed with a High Standard All-in-One Fluorescence Microscope (Biorevo BZ-9000; Keyence Japan). The images obtained were merged and analysed by use of the accompanying image analysis software with Biorevo BZ-9000.

### Immunohistochemistry for p16^INK4a^ Expression

After immersion in 0.3% H_2_O_2_/methanol, sections were preincubated with 10% normal goat serum. After antigen retrieval, tissues were incubated overnight at 4°C purified mouse anti-human p16 (BD Biosciences Pharmingen, San Diego, CA, USA) at a 1:300 dilution. The slides were subsequently incubated with biotinylated goat anti-rabbit antibody for 1 h at room temperature, and then avidin–peroxidase, and then visualized with DAB.

### Double-Label Immunofluorescence

We also carried out double-label immunofluorescence staining for 53BP1 and Ki67 expression to clarify the association between type of 53BP1 expression and cyclingecells. For double staining, tissues were incubated with a mixture of rabbit anti-53BP1 and monoclonal mouse anti-Ki67 (MIB-1; DakoCytomation) antibodies at 1:50 dilutions, and subsequently incubated with a mixture of Alexa Fluor 488-conjugated goat anti-rabbit and Alexa Fluor 546-conjugated goat anti-mouse antibodies. Specimens were counterstained with DAPI-I (Vysis), and visualized and photographed with a High Standard All-in-One Fluorescence Microscope (Biorevo BZ-9000; Keyence Japan). Signals were analysed at a ×1000 magnification.

### Statistical Analysis

The Jonckheere–Terpstra test was used to assess associations between the type of HPV ISH signal (negative, diffuse, mixed diffuse and punctate, and punctate) or the type of 53BP1 expression (stable, low DDR, high DDR, and large NF) and histological grade of cervical neoplasia. Associations between the types of 53BP1 expression and HPV ISH signal were also assessed with the Jonckheere–Terpstra test. The PHREG procedure in the SAS 8.2 software (SAS Institute, Cary, NC, USA) was utilized for calculation. All tests were two-tailed, and a *P*-value of <0.05 was accepted as statistically significant.

## Results

### HR-HPV ISH Signal in Cervical Lesions

**Figure 2:**
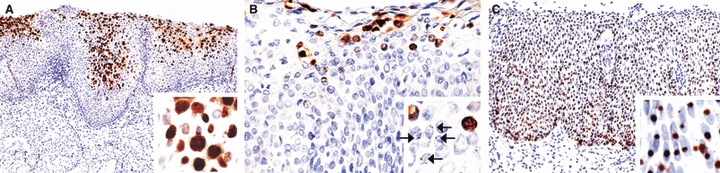
*In-situ* hybridization signals of high-risk human papillomavirus in cervical lesions. Signal types were classified as diffuse [**A**, cervical intraepithelial neoplasia (CIN)1], mixed (**B**, CIN2), or punctate (**C**, CIN3). Arrows in the inset of **B** indicate punctate signals.

The ISH results for each type of cervical lesions are summarized in Table 1. Representative types of ISH signals are illustrated in Figure 2. All normal cervical epithelium cases were negative for HPV ISH signals. Of the 16 CIN1 cases, nine (56.3%) expressed no signals, three (18.8%) expressed the diffuse type, and two (12.5%) were of the mixed and punctate type. Of the 15 CIN2 cases, three (20%) were of the negative and diffuse type, three (20%) were of the mixed type, and six (40%) were of the punctate type. Of the 24 CIN3 cases, only one (4.2%) was negative, three (12.5%) were of the diffuse type, nine (37.5%) were of the mixed type, and 11 (45.8%) were of the punctate type. All of the 14 SCC cases were positive for HPV ISH signal: four (28.6%) were of the mixed type and nine cases (64.3%) were of the punctate type, whereas only one (7.1%) expressed the diffuse type. Statistical analysis revealed significant associations between type of HPV ISH signal and type of cervical lesion (*P* < 0.0001). The incidence of mixed and punctate types significantly increased in the order of normal, CIN1, CIN2, CIN3, and SCC.

**Table 1:** Summary of high-risk human papillomavirus (HPV) *in-situ* hybridization (ISH) signal in the subjects used in this study

			Type of HPV ISH signal, no. (%)			
			
	*n*	Mean age [years (range)]	Negative	Diffuse	Mixed	Punctate
Normal	11	42 (36–45)	11 (100)	0	0	0

CIN1	16	39 (18–68)	9 (56.3)	3 (18.8)	2 (12.5)	2 (12.5)

CIN2	15	36 (27–50)	3 (20)	3 (20)	3 (20)	6 (40)

CIN3	24	38 (28–62)	1 (4.2)	3 (12.5)	9 (37.5)	11 (45.8)

SCC	14	57 (31–76)	0	1 (7.1)	4 (28.6)	9 (64.3)

CIN, cervical intraepithelial neoplasia, SCC, squamous cell carcinoma.

*P* < 0.0001, Jonckheere–Terpstra test.

### 53BP1 Expression in Cervical Lesions

**Figure 3:**
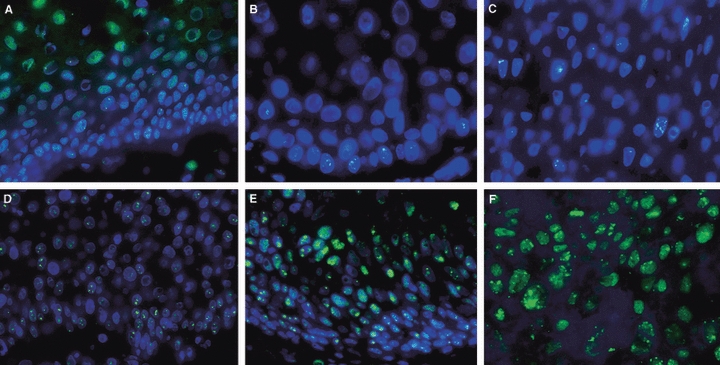
Immunofluorescence for p53-binding protein 1 (53BP1) expression in cervical lesions. Normal cervical epithelium (**A**) expressed the stable type of staining, with rare, one or two nuclear foci (NF), whereas cervical intraepithelial neoplasia (CIN)1 (**B**) and CIN2 (**C**) occasionally expressed three or more NF in dysplastic cells at the basal layer. CIN3 showed several discrete NF (**D**) with occasional large foci (**E**) throughout the epithelium. Squamous cell carcinoma (**F**) showed several discrete NF including large foci.

The results of the immunofluorescence staining patterns for 53BP1 in cervical lesions are presented in Table 2, and typical examples are depicted in Figure 3. Quantitation of the signals revealed immunopositivity in 4877 nuclei (443.4 nuclei per case) in normal cervical epithelium, 10 483 nuclei (655.2 nuclei per case) in CIN1, 9976 nuclei (665.1 nuclei per case) in CIN2, 14 953 nuclei (623.0 nuclei per case) in CIN3, and 7979 nuclei (569.9 nuclei per case) in SCC. In the normal cervical epithelium, 95.8% of nuclei showed the stable type, and only 4.3% of nuclei showed the DDR type. In CIN1, 73.4% of nuclei showed the stable type, and 25.2% and 1.4% of nuclei showed the DDR and large NF types, respectively. In CIN2, 58.4% of nuclei showed the stable type, and 38.1% and 3.5% of nuclei showed the DDR and large NF types, respectively. In CIN3, 33.7% of nuclei showed the stable type, and 58.5% of nuclei showed DDR types, including 37.5% with the high DDR type and 7.8% with the large NF type. Finally, in SCC, only 18.3% of nuclei showed the stable type, and 65.6% of nuclei showed DDR types, including 49.8% with the high DDR type and 16.2% of nuclei with the large NF type. The statistical analysis revealed that the histological type of cervical neoplasm was significantly associated with type of 53BP1 expression (*P* < 0.0001).

**Table 2:** Results for type of p53-binding protein 1 (53BP1) expression in cervical lesions by immunofluorescence

			Type of 53BP1 expression (%)			
			
	*n*	Counted nuclei	Stable	Low DDR	High DDR	Large foci
Normal	11	4877	95.8	3.1	1.2	0.0

CIN1	16	10 483	73.4	15.0	10.2	1.4

CIN2	15	9976	58.4	19.0	19.1	3.5

CIN3	24	14 953	33.7	21.0	37.5	7.8

SCC	14	7979	18.3	15.8	49.8	16.2

CIN, cervical intraepithelial neoplasia; DDR, DNA damage response; SCC, squamous cell carcinoma.

*P* < 0.0001, Jonckheere–Terpstra test.

### Association Between 53BP1 NF and HR-HPV ISH Signals in Cervical Epithelium

Associations between types of 53BP1 expression and HR-HPV ISH signal in the cervical epthelium are summarized in Table 3. In the cervical epithelium with negative HR-HPV ISH signal, 78.5% of nuclei showed the stable type, and 20.5% of nuclei showed the DDR type. In the cervical epithelium with the diffuse type of HR-HPV ISH signal, 55.6% of nuclei showed the stable type, and 42.1% and 2.4% of nuclei showed the DDR and large NF types, respectively. In the cervical epithelium with the mixed type of HR-HPV ISH signal, 39.3% of nuclei showed the stable type, and 51.2% and 9.6% of nuclei showed the DDR and large NF types, respectively. Finally, in the cervical epithelium with the punctate type of HR-HPV ISH signal, 35.1% of nuclei showed the stable type, and 54.3% of nuclei showed DDR types, including 35.9% with the high DDR type and 10.6% with the large NF type. The statistical analysis revealed that the type of HR-HPV ISH signal in cervical neoplasms was significantly associated with type of 53BP1 expression (*P* < 0.0001). The incidence of the DDR and large NF types significantly increased in the order of negative, diffuse, mixed and punctate types of HR-HPV ISH signal.

**Table 3:** Association between types of p53-binding protein 1 (53BP1) expression and human papillomavirus (HPV) *in-situ* hybridization (ISH) signal in cervical epithelium

		Type of 53BP1 expression (%)			
		
Type of HPV ISH signal	*n*	Stable	Low DDR	High DDR	Large foci
Negative	24	78.5	12.6	7.9	1.0

Diffuse	10	55.6	17.7	24.4	2.4

Mixed	18	39.3	20.4	30.8	9.6

Punctate	28	35.1	18.4	35.9	10.6

DDR, DNA damage response.

*P* < 0.0001, Jonckheere–Terpstra test.

**Figure 4:**
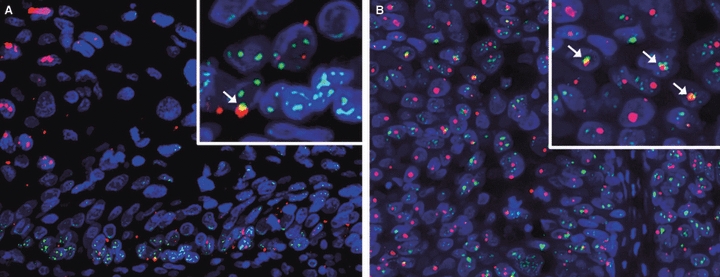
Dual immunofluorescence for p53-binding protein 1 (53BP1) expression (green) and *in-situ* hybridization (ISH) signals of high-risk human papillomavirus (red). The distribution of 53BP1 nuclear foci (NF) was the same as that of the punctate type of ISH signal in both cervical intraepithelial neoplasia (CIN)1 with the mixed type (**A**) and squamous cell carcinoma with punctate type (**B**) of ISH signal. However, co-localization of 53BP1 NF and the punctate type of ISH signal was very rare. Arrows indicate co-localization of 53BP1 NF and the punctate type of ISH signal.

The results of the dual immunofluorescence staining for 53BP1 expression and ISH to detect HR-HPV are shown in Figure 4. In CIN showing the mixed type of HR-HPV ISH signal, 53BP1 NF and the punctate type of HR-HPV ISH signal were similarly distributed in the dysplastic cells at the basal portion in cervical epithelium. Also in SCC showing the punctate type of HR-HPV ISH signal, 53BP1 NF and the punctate type of HR-HPV ISH signal were similarly distributed throughout the cancer cells. However, co-localization of 53BP1 NF and the punctate type of HR-HPV signal was very rare.

### Association Between 53BP1 and p16^INK4a^ Expression in Cervical Lesions

**Figure 5:**
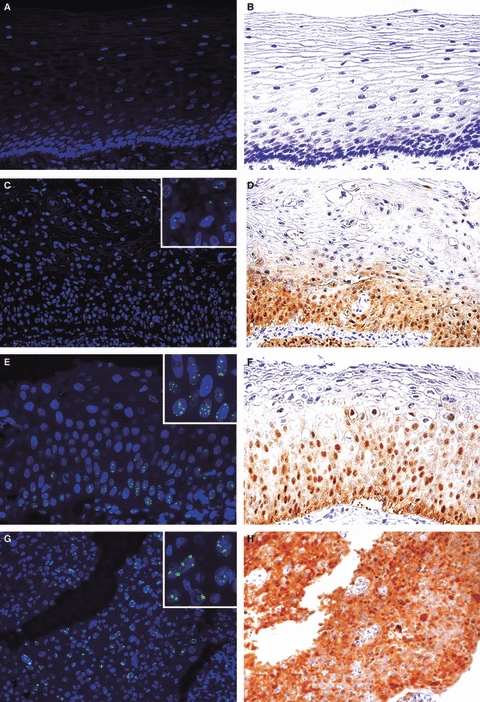
Comparison of p53-binding protein 1 (53BP1) (**A**,**C**,**E**,**G**) and p16^INK4a^ expression (**B**,**D**,**F**,**H**) in semi-serial sections of cervical lesions. The distribution of 53BP1 nuclear foci was similar to that of p16^INK4a^ overexpression in normal (**A**,**B**), cervical intraepithelial neoplasia (CIN1) (**C**,**D**), CIN2 (**E**,**F**), and squamous cell carcinoma (**G**,**H**).

The comparison of 53BP1 expression and p16^INK4a^ expression in cervical lesions is shown in Figure 5. The distribution of 53BP1 NF matched p16^INK4a^ overexpression in each cervical lesion.

### Double-Label Immunofluorescence Staining for 53BP1 and Ki67

**Figure 6:**
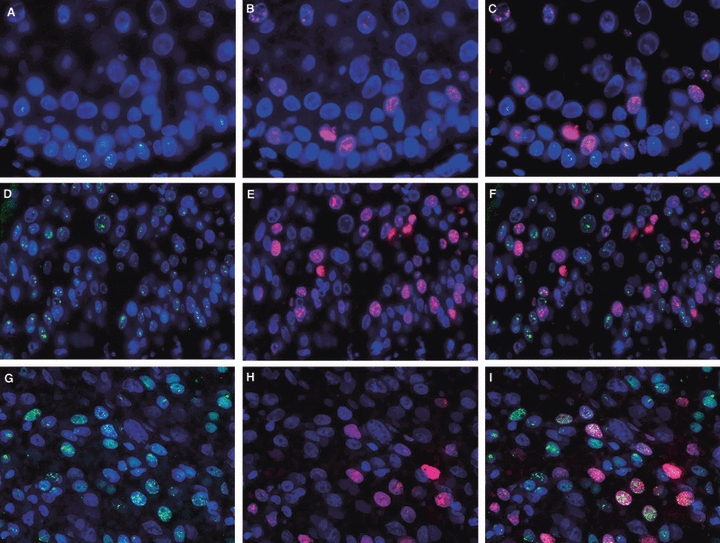
Double-label immunofluorescence staining for p53-binding protein 1 (53BP1) (green) and Ki67 expression (red) in cervical lesions. Cervical intraepithelial neoplasia (CIN)1 showed no or a few nuclear foci (NF) of 53BP1 immunoreactivity and little Ki67 nuclear staining, mainly at the basal layer (**A**–**C**). CIN3 showed several 53BP1 NF that were independent of Ki67 nuclear staining in cancer cells (**D**–**F**). Squamous cell carcinoma showed nuclei with both 53BP1 NF and Ki67 immunoreactivity (**G**–**I**), suggesting disruption of the DNA damage response pathway.

CIN1 expressed the stable or low DDR type of 53BP1 immunoreactivity, and little Ki67 nuclear staining, mainly at the basal layer (Figure 6). In contrast, a high level of the DDR and large NF type of 53BP1 expression and several nuclei showing Ki67 staining were observed in CIN3 and SCC cells. Furthermore, double staining for 53BP1 and Ki67 demonstrated that NF of 53BP1 immunostaining were not co-localized with Ki67-positive dysplastic cells in CIN3, whereas 53BP1 NF-positive cells frequently expressed Ki67 nuclear staining in SCC.

## Discussion

Ionizing radiation (IR) effectively induces DSBs in normal cells and activates DDR pathways to maintain genomic integrity. Although non-irradiated cells typically show diffuse 53BP1 nuclear staining, many 53BP1 NF are immediately induced after exposure to a low dose of IR.^13^ Schultz *et al.*^6^ showed that the number of 53BP1 NF induced by IR increased linearly with the dose of IR. On the other hand, we found that HeLa cells express occasional discrete 53BP1 NF without any genotoxic treatments, suggesting the occurrence of endogenous DSBs in cancer cells. One manifestation of GIN is induction of endogenous DSBs;^17^ thus, the level of 53BP1 NF formation can be considered to be a cytological marker for GIN. The present study has clearly demonstrated that the number of discrete NF (DDR type) of 53BP1 in cervical cells increases with tumour progression, as seen by the normal–CIN1–CIN2–CIN3–SCC sequence. The number of discrete NF in the DDR type of 53BP1 NF in the cervical epithelium seems to increase in precancerous lesions, and the high DDR type and large NF type of 53BP1 immunoreactivity are predominantly observed in SCC. Similar results, showing the differences in 53BP1 expression patterns during carcinogenesis, were obtained in our previous studies on thyroid and skin tumours resected from patients.^15^,^16^ Therefore, we propose that immunofluorescence analysis of 53BP1 expression can be a useful tool with which to estimate the level of GIN as well as the malignant potential of several human tumours.

The value of p16^INK4a^ as a surrogate marker of HR-HPV and CIN has been well established in recent years, with studies showing both increased immunoexpression of p16^INK4a^ in dysplastic cervical epithelium and a positive correlation with HR-HPV infection and the degree of CIN.^24^–^30^ Cervical SCC develops through a multistep process that involves replication stress. An important occurrence in cervical carcinogenesis is deregulated expression of the HR-HPV oncogenes *E6* and *E7*. Several risk factors for cervical neoplastic progression are likely to contribute to viral oncogene deregulation, particularly integration of HR-HPV into the host genome.^31^,^32^ We found a significant association between the type of HR-HPV ISH signal and type of 53BP1 expression in the cervical epithelium. The distribution of 53BP1 NF was similar to that of the punctate type of ISH signals in both CIN and SCC, suggesting that viral integration could induce endogenous DSBs through the induction of GIN in the host genome. Furthermore, in cervical lesions, the distribution of 53BP1 NF was identical to that of p16^INK4a^ overexpression; this represents a byproduct of viral infection, and suggests an association between the presence of GIN and replication stress that allows enhancement of cell proliferation and avoidance of cell death. As co-localization of 53BP1 NF and the punctate type of HR-HPV ISH signal was rare, most viral integrated sites might have been bypassed by the host's DDR machinery, and integrants may have a growth advantage over others in a mixed population of cervical cells.

This study also demonstrated little co-localization of the DDR type of 53BP1 expression and Ki67 nuclear staining as a marker for cycling cells in precancerous lesions. An intact DDR pathway is activated, leading to cell cycle arrest by p53 activity, so precancerous/dysplastic cells may still preserve DDR function, at least partially, and this may induce sporadic regression of dysplasia. In contrast, cancer cells showing 53BP1 NF were frequently co-localized with Ki67 staining, suggesting a disruption of the DDR pathway that subsequently leads to an irreversible malignant transformation. Taken together, these findings indicate that GIN may have already occurred at the precancerous stage during cervical carcinogenesis, and that increasing GIN based on a disrupted DDR may allow further accumulation of other genomic alterations, causing progression to invasive cancer through acceleration of cell growth/replication stress. Further research is needed on the molecular mechanism of the uncoupling of cell cycle progression in the face of increasing severity of DNA damage at the advanced phase of carcinogenesis.

In summary, this study has demonstrated a number of 53BP1 NF in cervical lesions resected from patients that were similar to those found in irradiated cells with a DDR pathway activated to eliminate DSBs, suggesting the occurrence of endogenous DSBs. GIN seems to be induced at the precancerous stage through integration of the HR-HPV genome into the host cell genome. Furthermore, invasive cancers exhibited 53BP1 NF in cycling cells, which suggested that the disrupted DDR subsequently led to further amplification of the genomic injury. A recent study on immunohistochemical detection of DDR-associated molecules, including 53BP1, suggests that GIN is an early event that occurs in pulmonary hyperplasia prior to changes in the p53 tumour suppressor gene during lung carcinogenesis in patients, suggesting that GIN may serve as a causative link between precancer and cancer.^33^ Thus, measurement of GIN, a hallmark feature of solid tumours that is implicated in both the initiation and progression of cancers, may serve as a valuable molecular marker of malignant potential. Our recent study also demonstrated that the detection of 53BP1 NF by immunofluorescence can be a useful histological marker with which to estimate the malignant potential of thyroid and skin tumours.^15^,^16^ Thus, we propose that immunofluorescence analysis of 53BP1 expression can be a useful tool with which to estimate the level of GIN and, simultaneously, the stage of cancerous progression of uterine cervical lesions.

## Abbreviations

53BP1: p53-binding protein 1
CIN: cervical intraepithelial neoplasia
DAB: diaminobenzidine
DAPI-I: 4′,6-diamidino-2-phenylindole dihydrochloride
DDR: DNA damage response
DSB: double strand break
GIN: genomic instability
HPV: human papillomavirus
HR-HPV: high-risk human papillomavirus
IR: ionizing radiation
ISH: *in-situ* hybridization
NF: nuclear foci
pRb: retinoblastoma protein
SCC: squamous cell carcinoma
